# Myofascial System and Physical Exercise: A Narrative Review on Stiffening (Part II)

**DOI:** 10.7759/cureus.76295

**Published:** 2024-12-24

**Authors:** Saverio Colonna, Fabio Casacci

**Affiliations:** 1 Rehabilitation Medicine, Spine Center, Bologna, ITA; 2 Research and Development, Osteopathic Spine Center Education, Bologna, ITA

**Keywords:** fascial contractility, flexion relaxation phenomenon, hill’s three-element model, human resting myofascial tone, miofascial system, muscle strengthening exercise, myofascial pain, therapeutic fascial strengthening

## Abstract

In the past two decades, interest in the fascial system has exponentially increased, particularly manual treatment and stretching exercises. The fascia's fundamental role remains the transmission of tensions, although this function can be impaired due to excessive or reduced stiffness. This second part of the work outlines the basic principles concerning the importance of appropriate and balanced fascial stiffness for correct postural and functional maintenance of the human body. Additionally, the limited studies available in the literature are reviewed, with a focus on therapeutic exercises aimed at increasing fascial system stiffness. The article addresses how fascia develops the ability to contract to maintain a physiological tension referred to as human resting myofascial tone. Additionally, it discusses the most recognized tools for assessing fascial tension: myotonometry and shear wave elastography. The final section is dedicated to presenting the current literature on the relationship between physical exercise and fascial stiffness.

## Introduction and background

This article concludes the presentation of the therapeutic approach to fascia, initiated in part I of this work [1], by integrating recent research findings with previous results. The first part of the study [1], starting with histology and progressing to physiology and intervention methods through stretching, presents a new perspective on the therapeutic approach to issues characterized by excessive myofascial stiffness as their etiopathogenesis. In this article, we address how to better interpret fascial dysfunctions, focusing on the opposite, often overlooked, dysfunction: reduced fascial stiffness.

As reported and cited in the specific paragraph, several studies have evaluated muscle responses to exercise, but research has predominantly focused on the response of muscle myofibrils rather than the connective tissue component, namely the fascial part. The proliferative response of muscle cells to exercise, either in series or in parallel, leads to specific muscular adaptations. Extending this type of response to the passive fascial component without appropriate investigation may result in significant errors. Understanding which responses of the fascial system, when and how, lead to increased stiffness can facilitate therapeutic approaches in various pathologies.

Revision of the fascial dysfunction concept

As mentioned previously in part I, the fascia's main role is to transmit mechanical tension. Regarding force transmission, an erroneous belief persists that alterations in this function, defined in osteopathy as somatic dysfunction [2], are typically attributed to increased rigidity (stiffness) that causes limited joint movement, which is referred to as the motion barrier [2-4].

These dysfunctional connective system models can lead to, for example, opposite foot conformations: excessive tension in the myofascial systems causes an accentuation of the foot's medial arch (a condition known as pes cavus) [4], whereas insufficient tension causes a reduction in the arch, which is known as pes planus (flat foot) [5]. Thus, it is a mistake to focus only on altering excessive stiffness [3] and ignore reduced stiffness [6].

## Review

Fascial laxity dysfunction

Fascial laxity dysfunction is a pathological or functional condition characterized by reduced tension or insufficient support provided by the fascial system, resulting in decreased mechanical stability, impaired force transmission, and potential joint or tissue instability. Pathological reductions in connective system stiffness can be systemic, affecting the entire body, as in Ehlers-Danlos syndrome, Marfan syndrome, or hypermobility syndrome, or they can be localized, as seen in inguinal hernia, abdominal hernia, or lumbar hernia [6].

The lack of adequate fascial system stiffness, remembering that the fascia's primary role is tension transmission, can be secondary to trauma: excessive elongation with capsulo-ligamentous tearing can lead to a loss of joint stability or it can be related to prolonged abnormal postural attitudes that may result in localized loss of physiological stiffness. In traumatic cases, insufficient fascial stiffness is often considered a consequence of ankle sprains, whereas in atraumatic cases, the lumbar and cervical spine may be investigated, as alterations in sagittal plane curves can lead to a reduction in the physiological lumbar curve (hypolordosis).

If numerous therapeutic approaches for low back pain (LBP) aim to reduce the stiffness of the fascial system, which plays a key role in joint stability, how can we reconcile the effectiveness of these treatments with lumbar instability as the primary cause of such issues? [7].

**Figure 1:**
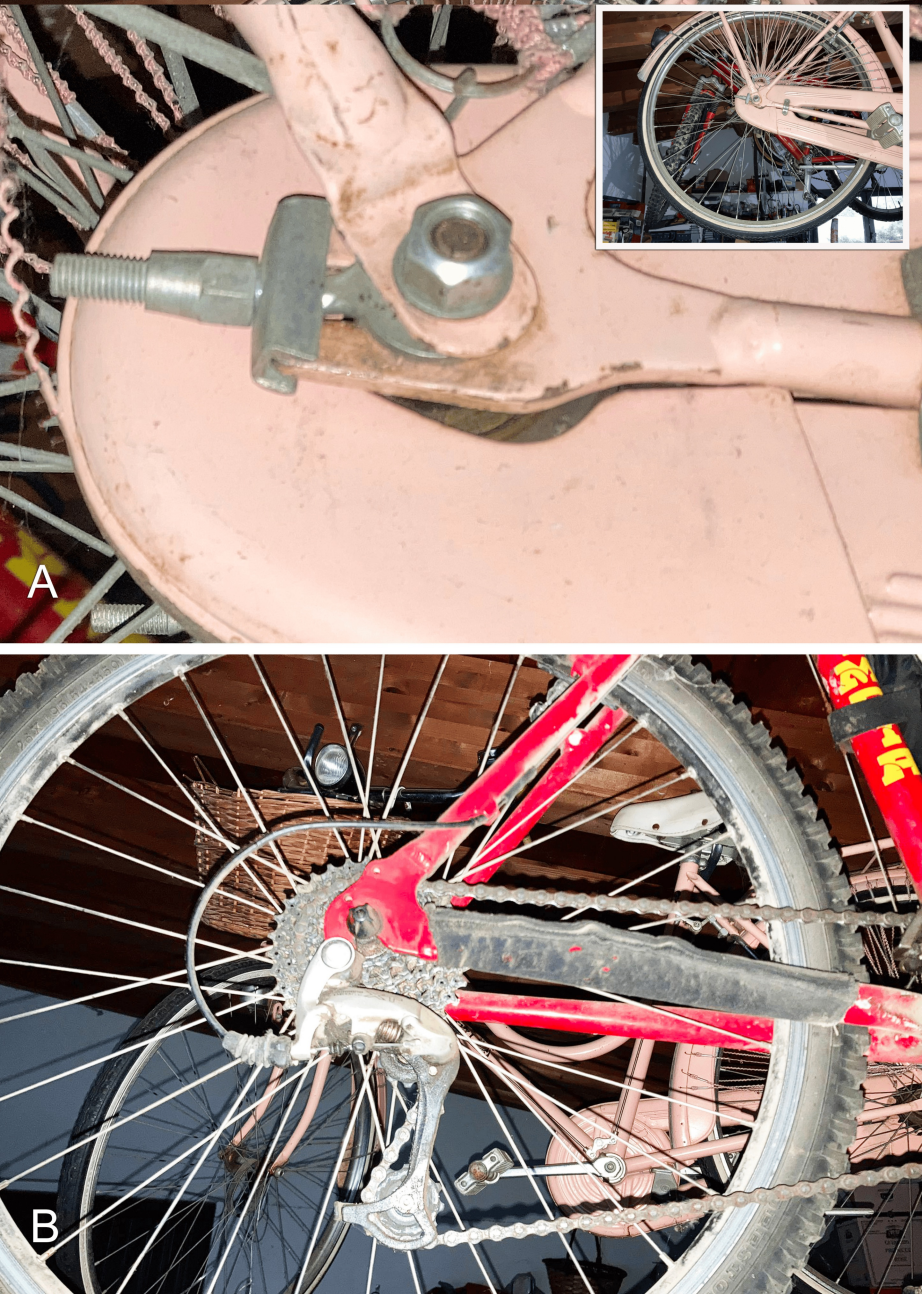
Bicycle drive chain tensioning models (A) Old model. (B) Recent model. Image credit: Author Saverio Colonna

Literature data on techniques to increase fascial stiffness, unlike methods to stretch the fascia, are sparse if not entirely absent. The fascial system can be compared to a well-known mechanical tension transmission system: the bicycle chain. An altered function of the chain, which can be defined as dysfunction, is excessive tension or, conversely, reduced tension. Therefore, this method of locomotion has a simple system for tensioning and releasing the chain connected to the rear wheel (Figure 1).

The same logic should be applied to the human myofascial system, as it is similarly a tension transmission system. When evaluating musculoskeletal segments, osteopaths often assign dysfunction by default to the side of restriction, although this may not be biomechanically logical [2]. An example is the straightening of the lumbar spine, which is a vertebral flexion dysfunction that involves restricted extension mobility and facilitated flexion [8], with a positive osteopathic spring test [9]. It is not entirely correct to attribute the result of this test solely to increased stiffness of the anterior fascial systems, as the primary cause may be the loss of stiffness in the posterior fascial systems.

**Figure 2:**
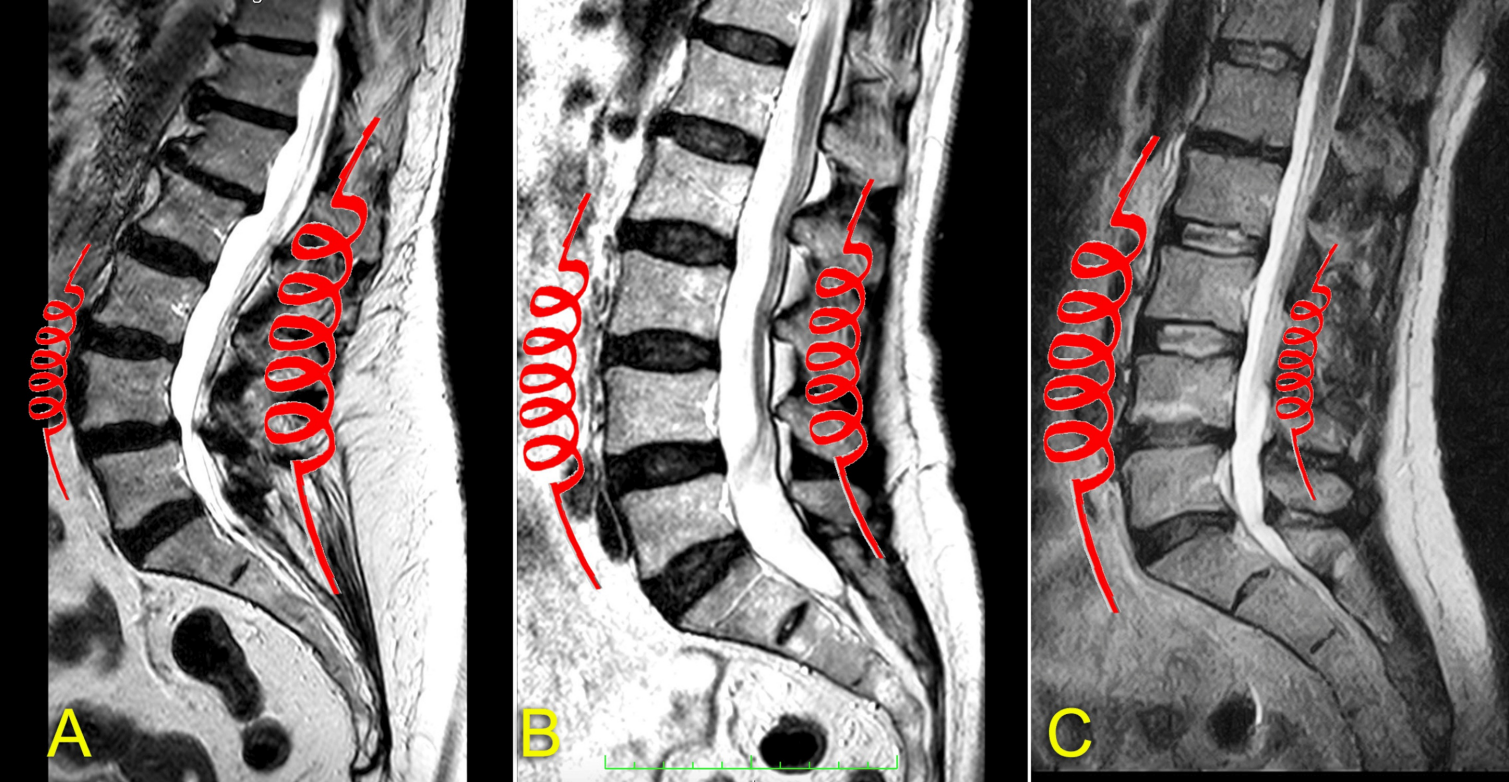
Sagittal MRIs of three models of anterior and posterior fascial stiffness balance in the lumbar spine (A) Posterior predominance. (B) Balance. (C) Anterior predominance. Image credit: Author Saverio Colonna

Consequently, the correct lumbar pathology treatment when a reduction in the lumbar lordotic curve is present requires lengthening the anterior fascial system, where there is excessive stiffness, and shortening the posterior one, where there is reduced stiffness (Figure 2C).

Increasing physiological stiffness does not mean altering the fascia’s structural composition by, for example, reducing the amount of hyaluronic acid, which would lead to increased density and compromise the ability to glide. Therefore, the question arises: Does the fascial system have the inherent ability to physiologically increase stiffness? To answer this question, we must explore some physiological fascial behaviors.

Fascial viscoelasticity

The fascia has the capacity for both elastic and plastic deformation in a non-linear fashion, influenced by the magnitude, duration, and speed of the applied load [10]. This viscoelastic property arises from the interrelationship between the connective tissue's structure, composition, and water content [11].

Research has shown that fibrils and the interfibrillar matrix function as coupled viscoelastic systems, exhibiting qualitatively distinct responses to mechanical deformation based on the cross-linking within collagen’s molecular structure [12]. Additionally, changes in tension within the connective tissue prompt an immediate reorganization of the fibroblast cytoskeleton, leading to modifications in tissue stiffness and viscosity [13].

Notably, an intrinsic viscoelastic property of the myofascia has been identified that operates independently of nervous system activity, as indicated by silent electromyography (EMG) patterns [14,15]. Known as human resting myofascial tone (HRMT) [14], this property is governed by molecular interactions between actomyosin filaments in myofibroblastic cells and myosarcomeric units.

This tone plays a crucial role in maintaining postural stability with minimal energy expenditure, in contrast to neuromotor activation, which requires higher levels of muscle tone for stabilization. This aligns with findings from studies in pathological conditions, where sustained static loading induces viscoelastic creep in connective tissue, leading to temporary changes in neuromuscular activity (such as muscle spasm and hyperexcitability), with the intensity of these changes being directly proportional to the load magnitude [16]. Alterations in normal HRMT levels can impact the tension in surrounding fascial structures, potentially affecting joint mobility, movement control, and postural stability - all factors linked to various musculoskeletal conditions and dysfunctions [15]. These changes may also contribute to a shift in the colloidal consistency of the ground substance, moving it toward a more solid state. Thixotropy is the property of a substance to decrease its viscosity when agitated or stirred and then to solidify when left to rest [17].

Fascial contractility

The fascial system can increase its intrinsic stiffness both through modification of its architecture, as we saw in the previous section, and through actual contraction. Cells such as fibroblasts [18] and myofibroblasts [19] exhibit a marked contractile ability. Myofibroblasts, under certain conditions, demonstrate a contractile capacity four times greater than fibroblasts [20]. This contraction appears to primarily develop to bring the edges of wounds closer together during the healing process. The fascia is capable of contracting in a manner similar to smooth muscle [21], independently of skeletal muscle activity. This is related to the presence of specific cells in the fascia called myofibroblasts, which have a particular histological structure, representing an intermediate form between a muscle cell and a fibroblast. Myofibroblasts have the ability to contract via intracellular actin, and for this reason, these cells are known as alpha-smooth muscle actin (α-SMA) [22,23]. The behavior and contraction of myofibroblasts are highly responsive to oxygen levels, vasoactive peptides, autonomic activity, proinflammatory cytokines, and surrounding mechanical tension [24]. Given their complexity and relatively recent discovery, many aspects of myofibroblasts remain poorly understood.

Similar to smooth muscle cells, myofibroblasts appear to exhibit various phenotypes with significant differences in contractile behavior [25].

Myofibroblasts can generate sustained isometric contractions [23], which are transmitted to the extracellular matrix (ECM) through focal adhesions and associated stress fibers. Schleip et al. suggest, based on observations and laboratory studies on the rat soleus muscle, that the fascial layer with the highest concentration of myofibroblasts, and thus contractile ability, is the perimysium, especially in tonic muscles [26].

Perimysial myofibroblasts respond rapidly to alterations in mechanical stimulation, resulting in significant changes in passive myofascial stiffness. This likely influences overall resting muscle tone and musculoskeletal dynamics by locally redistributing mechanical force and segmental neurological influence on somatic motoneurons [26]. Dysfunction in this system can lead to altered myofascial tone, diminished neuromuscular coordination, musculoskeletal disorders, and pain syndromes [6].

The etiopathogenetic involvement of these structures may be relevant to a range of conditions associated with increased passive muscle stiffness (e.g., torticollis, muscular fibrosis, Parkinsonian rigidity, ankylosing spondylitis, and chronic neck and/or back pain due to persistent muscle stiffness), as well as conditions involving reduced myofascial stiffness, such as peripartum pelvic pain due to pelvic instability, and LBP related to segmental spinal instability or fibromyalgia [5].

Spinal stability and fascial systems

Spinal stability has been associated with trunk stiffness [27], as the elastic stiffness of trunk musculature is considered the spine’s primary stabilizing mechanism [28,29]. When the fascial system alone does not develop adequate passive tension, a compensatory tension system is likely prompted through active muscle contraction.

Shirley and Lee's findings suggest that people with LBP exhibit reduced posteroanterior spinal mobility and have greater muscle activity compared to people without pain [30]. Muscle activation could either be a consequence of the pain or a mechanism to increase vertebral stability. Accordingly, Lee et al. reported that a voluntary maximum contraction of 10% of the spinal erector muscle in asymptomatic individuals produces an 11.8% average increase in spinal stiffness [31].

Flexion relaxation phenomenon

An example of the relationship between reduced fascial stiffness and compensatory muscle activation is the flexion relaxation phenomenon (FRP). FRP is the myoelectric silence recorded via EMG in asymptomatic individuals’ erector spinae (ES) muscles during full trunk flexion in the standing position [32,33]. In the fully flexed standing posture, the weight of the upper body (trunk, upper limbs, and head) is primarily supported by an extension moment (the torque produced by the spine in response to the tendency of the body's center of mass to flex the spine) that is passively generated by spinal ligaments, intervertebral discs, and the passive components of the extensor muscles’ fascial units [34].

The absence of EMG silencing indicates a transfer of the extension moment production role from passive systems - likely belonging to the parallel fascial systems - to the deep muscles of the lumbar spine to achieve spinal stability and maintain balance [35].

Patients with back problems demonstrate laxity in passive structures and an altered neuromuscular activation pattern in spinal muscles, characterized by the absence of the FRP in the ES muscles.

Floyd and Silver [32] were the first to describe the lack of ES relaxation during trunk flexion in the standing position in patients with chronic LBP, a phenomenon later confirmed by more recent studies [36,37], which also included patients with radiculopathies [37].

Consequently, the FRP of the ES muscles has been utilized to assess patients with LBP and to monitor factors related to treatment interventions [38].

Initially observed in maximum trunk flexion in a standing position, FRP has also been observed in asymptomatic individuals during slumped sitting posture [32,39,40]. Cervical spine muscles are not exempt from EMG silence during flexion [41], and it has been suggested that a similar phenomenon may also involve the hamstring muscles [38]. The first scholar to evaluate not only the electrical activity of the paravertebral muscles but also that of the hamstrings during trunk flexion was Sihvonen in 1997 [42]. In this study, lumbar muscle activity ceased (i.e., FRP occurred) at an average lumbar flexion of approximately 79°, whereas the EMG activity of the hamstrings persisted longer, ceasing when near-complete lumbar flexion of 96.6% was reached.

**Figure 3:**
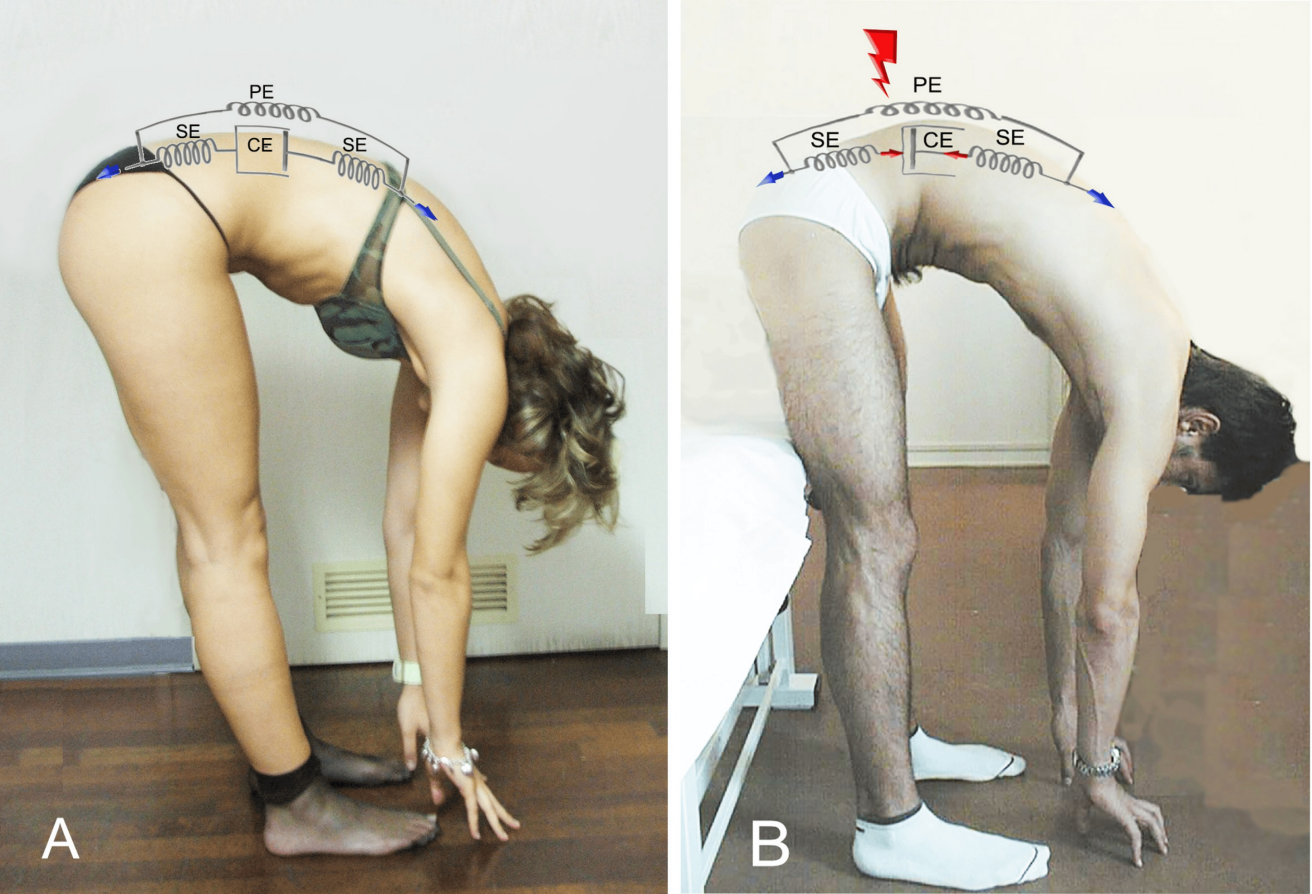
Schematic of the use of fascial systems in healthy and pathological subjects (A) Healthy subjects demonstrate a greater prevalence of using fascial systems in parallel during maximum trunk flexion. (B) Subjects with lumbar pain show a greater prevalence of using the muscular contractile system and related fascial systems in series to compensate for the reduced tension of the fascial system in parallel. PE, parallel element; SE, series element; CE, contractile element Image credit: Author Saverio Colonna

Returning to Hill's simplified subdivision of fascial component arrangement, presented in the first part of this work [1], we hypothesize that the parallel fascial component is responsible for developing the moment of force necessary to maintain trunk suspension during flexion without involving the fascial component in series, which is connected to the active part of the muscle. When this parallel system loses its containment capacity, the fascial system in series takes over by activating the contractile muscular component (Figure 3).

Fascial stiffness

Stiffness, defined as the ability to resist deformation when subjected to mechanical stress, is a mechanical property of tissues [43,44]. Stiffer tissues deform less than softer tissues [13]. Several factors need to be considered, including stress, which is the stimulus applied to the tissue, and strain, which represents the resulting deformation from the applied stress. Additionally, biological tissues are viscoelastic, possessing both solid and liquid components [45] that cause them to deform less if stress is applied rapidly [45].

Consequently, it follows that, to maintain physiological stiffness in fascial connective tissues, low-intensity tensile loads sustained over time are more damaging than higher loads applied for a shorter period.

It has been reported that tissue stiffness plays a crucial role in regulating cell division, maintaining tissue boundaries, directing cell migration, and guiding differentiation [46]. Therefore, it is essential to emphasize that an appropriate level of stiffness, achieved through external mechanical forces, is vital for maintaining homeostasis.

Instrumental evaluation of fascial stiffness

Because deformation and stress can vary significantly in vivo, measuring tissue stiffness presents a particular challenge. Currently, two primary noninvasive methods are commonly used to assess tissue stiffness: myotonometry, which is mainly employed to assess myofascial stiffness [47], and ultrasound elastography, which is a technique to evaluate both visceral organs (liver, kidneys, thyroid, prostate, or lymph nodes) [48] as well as nerves, tendons, ligaments, muscles, and fascia [49].

The measuring principle of the myotonometry is that the oscillations caused by multiple short pulses from a test probe, and their shapes, reflect the visco-elastic properties of the tissue such as tone and dynamic stiffness [50].

Myotonometry has been employed to evaluate various myofascial regions, including the ES [51,52], neck muscles [53], upper trapezius [54], vastus lateralis [55], plantar fascia [56], and many others [57].

Shear wave elastography (SWE) is a rapidly advancing ultrasound imaging technique designed to measure the mechanical and elastic properties of tissues. It complements traditional ultrasound methods, thus enhancing the evaluation and monitoring of traumatic and pathological conditions within the musculoskeletal system. Over the last two decades, sonoelastography has been increasingly utilized to assess soft-tissue elasticity, supplementing the data obtained from conventional gray-scale and Doppler ultrasound. Recently integrated into clinical imaging systems, SWE stands out for being more objective, quantitative, and reproducible compared to compression sonoelastography, with growing applications in musculoskeletal assessments. This technique employs an acoustic radiation force pulse sequence to produce shear waves that travel perpendicularly to the ultrasound beam, generating temporary tissue displacements. The velocity of these shear waves, measured at each pixel, directly corresponds to the shear modulus, a key parameter reflecting the tissue's elastic properties. SWE seamlessly integrates shear-wave images with standard B-mode ultrasound, producing quantitative, color-coded elastograms that maintain anatomical precision. Shear waves move faster in stiffer, contracted tissues and along the longitudinal axis of muscles and tendons. SWE has been employed to assess fascial stiffness in various body regions, including the plantar fascia [58], thoracolumbar fascia [59], ES muscle and lumbar multifidus muscle [60], hamstring [61], gastrocnemius fascia [62], gluteus medius and iliotibial band elasticity [63], and tensor fascia latae end gluteus maximus [64].

Physical exercise and fascial stiffness

Although exercise interventions are used to effectively treat pathological conditions and improve health outcomes, there remains a lack of knowledge about the physiological adaptations induced by different exercise regimens for specific modifications in tissue stiffness and thus appropriate exercise modalities.

The question we must ask is this: Are there methods that restore the physiological stiffness of fascial tissues?

The majority of the literature focuses on the stiffness of connective tissue related to tendons or the myotendinous junction [65]. However, few researchers have specifically examined fascial stiffness after exercise [66]. Regarding the metabolism of connective structures related to tendons, Magnusson et al. [65] describe how tendon collagen turnover is modified by physical exercise.

Mechanical load on the tendon structure leads to an increase in collagen protein synthesis, with a corresponding increase in collagen expression in humans and most of the animal kingdom [66,67].

**Figure 4:**
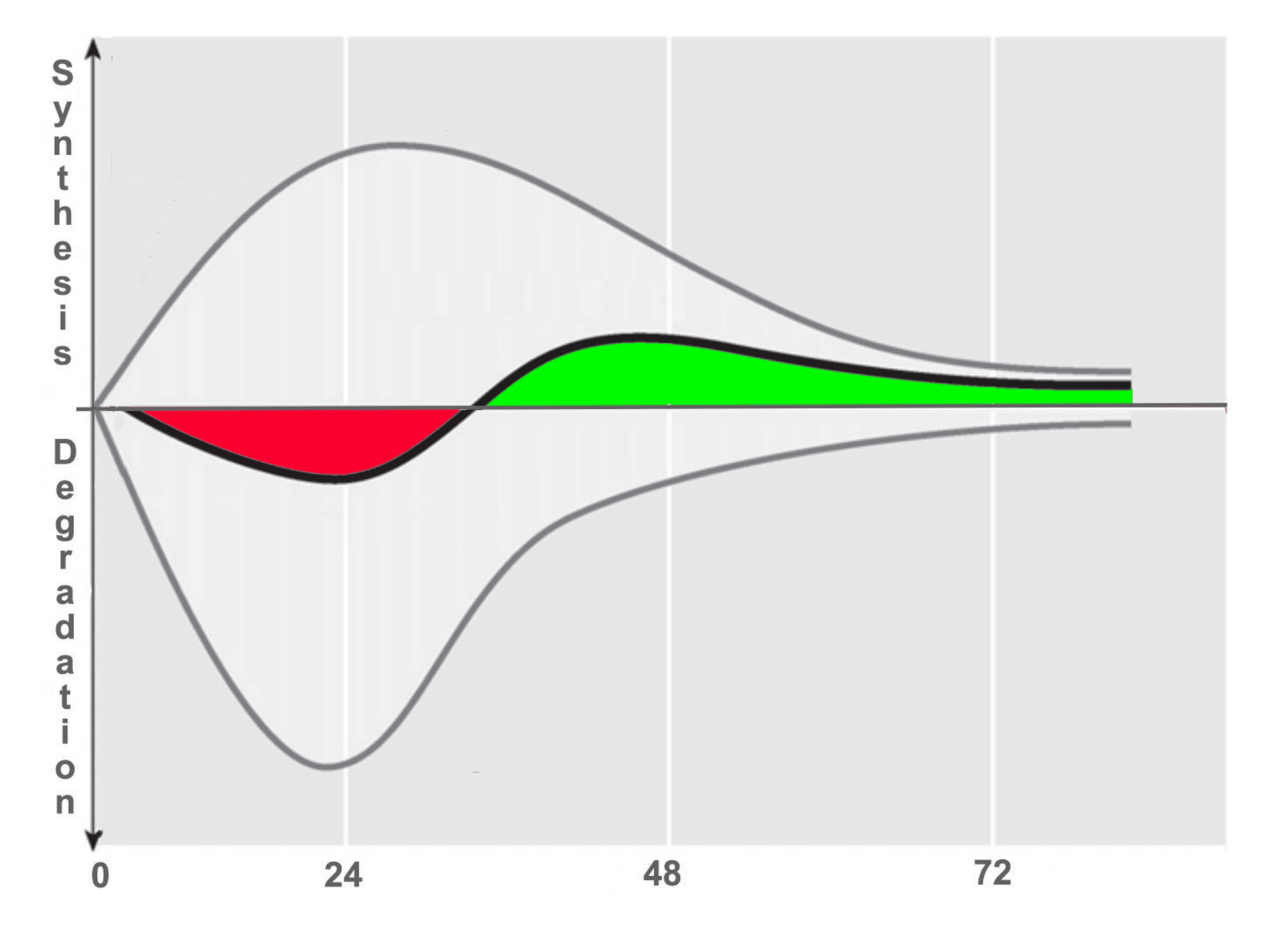
Turnover schematization of collagen after exercise The upper curve indicates that collagen synthesis in tendons increases after exercise begins (indicated on the graph as point 0). Stimulated fibroblasts also increase their rate of collagen degradation (represented by the lower curve), which in the first 24 hours is greater than the amount produced, thus resulting in a net degradation (represented by the negative red curve). The situation is then reversed, producing a net gain (represented by the positive green curve). To increase tendon strength, the proposed fascial fitness training through appropriate tissue stimulation should be used only one or twice per week. Illustration modified from the original by Magnusson et al. [50]. Image credit: Author Saverio Colonna

This elevated collagen expression is likely mediated by the tension exerted on the fibroblast, which can physiologically stimulate an increase of two to three times the regular amount of collagen, reaching a peak around 24 hours and remaining elevated for 70-80 hours following the exercise-induced stimulus, as illustrated in Figure 4 [68,69]. However, in response to exercise, both the anabolic phase of collagen synthesis and the catabolic phase of protein degradation increase [69], with degradation likely occurring at an initially greater rate than synthesis (Figure 4).

Levels of proteolysis markers, such as matrix metalloproteinases and collagen degradation fragments, increase in response to exercise [70] as part of the physiological adaptation to loading. Up to 18-36 hours after cessation of exercise, a net negative balance emerges in collagen levels, although this period shortens as the training state improves. The balance between degradation and production becomes positive for as many as 72 hours after exercise. This elevated turnover leads to a denser ECM within muscle fibers, resulting in significantly negative interstitial fluid pressure and subsequent muscle edema [71]. Similarly, studies by Hyldahl and Hubal [72] and Mackey et al. [73] suggest that changes in muscle biomechanical properties following unfamiliar eccentric contractions (ECC) are likely due to ECM remodeling.

Despite the ECM's crucial role in myofibrillar adaptation to exercise, its remodeling process remains surprisingly underexplored [68]. Madeleine et al. [74] proposed that changes in the muscle environment 24 hours after ECC might relate to increased muscle thickness. However, same authors’ findings showed no significant correlation between changes in muscle stiffness and thickness after eccentric exercise [54]. Interestingly, a prior study [75], employing a comparable training protocol, observed an increase in elbow flexor fascia thickness following eccentric exercise: the study proposed that this change might be attributed to fascial micro-injury and edema.

Previous studies have documented an increase in shear modulus and passive joint torque after eccentric exercise [76-77]. One study explored the changes in shear modulus and muscle stiffness as assessed by myotonometry, and while both methods indicated an increased muscle stiffness, there were no correlations between changes in both methods [78]. This indicates that the methods should probably not be used interchangeably. It is possible that myofascial edema associated with eccentric exercise [75] may underlie the differing responses observed between the two methodologies. SWE offers the advantage of providing a precise assessment of the depth at which the stiffness of the myofascial system is evaluated, a capability not available with myotonometry.

The findings of other studies [79] are consistent with recent discoveries, showing that intense eccentric loading of the knee flexors leads to increased stiffness of the deep fascia 1 hour and 3 days post-exercise, which correlates with the severity of delayed-onset muscle soreness (DOMS). These findings highlight the adaptability of extramuscular connective tissue to mechanical stress and support the theory that fascia contributes to the development of DOMS. As a result, fitness and conditioning professionals might benefit from implementing strategies focused on fascia to enhance recovery after workouts involving significant eccentric muscle activity.

On the other hand, some studies on the acute response of the myofascial system in the trapezius muscle following unaccustomed eccentric muscle contractions reported a decrease in stiffness [51], which was assessed using both myotonometry and SWE. In line with recent research, other findings have also demonstrated reductions in both the elastic modulus and dynamic stiffness [80-82]. A reduction in quadriceps stiffness, assessed via SWE, has been documented in athletes following an extreme mountain marathon [80]. Specifically, a notable decrease in the shear modulus of the quadriceps was observed immediately after the race compared to baseline levels. Partial recovery occurred within 48 hours post-race, affecting all muscle heads collectively and individually, including the rectus femoris, vastus medialis, and vastus lateralis [80].

It is suggested that training should be consistent and that a few minutes of targeted exercises performed once or twice per week are sufficient to stimulate collagen remodeling [65]. This renewal process takes between six months and two years, resulting in a flexible, agile, and resilient collagen matrix [50].

In their narrative review, Thomas et al. [66] assessed the long-term effects on tissue stiffness of certain exercise modalities, such as resistance, aerobic, and plyometric training as well as stretching. The authors conclude that specific tissue adaptations appear to be related to each exercise modality: resistance training increases tendon tissue stiffness, whereas the effects on muscles and muscle-tendon units provide mixed results. Plyometric training increases muscle tissue stiffness, but conclusive evidence for tendons could not be drawn [66].

Schoenrock et al. [83] indicate an HRMT increase in lumbar paraspinal muscles that were evaluated through myotonometry in subjects who were bedridden for approximately 60 days. The same was observed when these subjects resumed performing both uncontrolled and controlled physical activities, including jumping exercises [83]. However, different histochemical alterations were reported in both situations.

Santos et al.'s findings demonstrate an increase in vastus medialis stiffness, which was evaluated through elastography after 15 weeks of thrice-weekly training sessions using concentric and eccentric isokinetic modalities [84].

Another study found that a six-week training period with three weekly sessions did not modify triceps brachii stiffness [85]. This evaluation also used elastography in 20 healthy subjects.

The study by Rusu et al. aimed to compare the myotonometric assessment of muscle tone and fascial stiffness between two types of exercise programs: one focused on developing explosive strength and the other on maximum strength through isometric exercises [86]. However, the study's limitations include a sample size of only two subjects (one per program), no baseline measurements prior to the training programs for comparison with post-training assessments, and the absence of information regarding the duration of the training period.

The studies available in the literature show conflicting results, making it challenging to draw definitive conclusions on whether eccentric exercise leads to an increase or a decrease in myofascial stiffness.

An even more complex topic is whether the type of exercise performed specifically targets certain parts of the fascial system, such as those arranged in series or in parallel. To the best of our knowledge, no specific research has focused on the fascial component of muscles. However, a considerable body of literature provides indirect evidence regarding the proliferation of muscle sarcomeres arranged either in series or in parallel in response to exercise.

The physiological cross-sectional area (PCSA) is known to determine the maximum force a muscle can produce and its overall force-generating potential. Muscle volume, defined as the product of optimal muscle fiber length and PCSA, includes all sarcomeres arranged both in series and in parallel, serving as an indicator of the muscle's maximum power output [87]. During development, skeletal muscles exhibit significant adaptability, undergoing alterations in both their structural organization and the contractile characteristics of their components, influenced by growth [88,89] and training [90-92]. In general, during development, muscles primarily adapt to longitudinal bone growth and mechanical overload [93,94]. The influence of activity on muscle length appears to be strongly dependent on the length at which the muscle is held or the range of motion it undergoes. Studies on the low-pennate soleus muscles of rabbits subjected to low-frequency stimulation revealed an increase in the number of sarcomeres in series only when the muscles were maintained at an extended length [95,96]. Conversely, when muscles were held at a shorter length, a reduction in serial sarcomere number was observed [97]. This decrease was even greater than that seen in muscles immobilized at short lengths without activation. These findings suggest that contractile activity enhances the adaptation of sarcomeres in series. Additionally, activation appears to mitigate connective tissue accumulation typically caused by immobilization [98].

To our knowledge, no studies have examined the impact of isometric contraction training on the function or morphology of animal muscles. Consequently, the following sections focus exclusively on summarizing the effects of concentric and eccentric exercise interventions on muscle growth in animal models.

Concentric contractions involve muscle shortening while generating force. Activities such as uphill walking, which emphasize concentric contractions at shorter muscle lengths, have been shown to reduce the number of sarcomeres in series within the vastus lateralis and vastus intermedius muscles of rats [99]. Additionally, Lynn and Morgan [100] found that rats running uphill had fewer sarcomeres in series in the vastus intermedius compared to rats running downhill. The torque-angle curves of animals performing concentric exercises also demonstrated a narrower range compared to those of animals engaged in eccentric exercise.

Eccentric contractions performed across the full-length range of the tibialis anterior muscle led to an increase in the number of sarcomeres in series, whereas contractions limited to a smaller range of motion resulted in a reduction in sarcomere number compared to control muscles [101].

Eccentric exercises also promote the addition of sarcomeres arranged in parallel within muscle fibers, contributing to increases in both fiber cross-sectional area and PCSA. Additionally, when eccentric exercises are performed at longer muscle lengths, they yield greater increases in PCSA compared to those performed at shorter lengths [101]. These exercises enhance the length-force relationship by improving both the maximum force generation and the range of active force exertion. Franchi et al. reported that when resistance training with eccentric and concentric contractions is matched for maximum load or work, similar increases in muscle size are observed [102]. However, these hypertrophic adaptations seem to result from distinct structural changes, likely driven by differing myogenic and molecular responses associated with lengthening versus shortening contractions.

Muscle growth can occur through the addition of sarcomeres either in series or in parallel, depending on the type of training performed. Eccentric training, for instance, typically promotes the addition of sarcomeres in series, leading to an increase in muscle fiber length [103]. In contrast, concentric training encourages the addition of sarcomeres in parallel, resulting in greater fiber diameter [103]. These structural adaptations are crucial for enhancing muscle functionality in response to specific mechanical stimuli.

From the above, we can conclude that sarcomere proliferation in muscles is exercise-specific; therefore, the associated fascial proliferation is likely also exercise-specific. However, this remains a theoretical deduction.

Attempting to draw a parallel between the architectural arrangement of sarcomeres in series and parallel in exercised muscle and the organization of the connective tissue structure might be misleading, as the fascial-connective component may not follow the same logic.

Future research

Future research should aim to address several critical questions to enhance our understanding of therapeutic and preventive fascia exercises. A key area of inquiry involves elucidating the functional relationship between the tonic muscular and fascial systems. Researchers should investigate whether it is possible to clearly distinguish the fascial system in series from that in parallel, as well as identify which structures within the fascial system belong to each category. Understanding which parts of the parallel system have a purely stabilizing function is also essential. Moreover, the development of instrumental evaluation methods to differentiate the stiffness of fascial systems in series versus parallel represents an important research objective. Additional studies are needed to explore exercise modalities that can specifically reduce or increase the stiffness of the fascial system, either in parallel or in series. Finally, research should examine whether it is possible to target individual connective tissue components, such as collagen structures (as previously discussed for tendons) or myofibroblasts, to achieve precise therapeutic outcomes.

## Conclusions

The narrative review presented in this article leads readers to reconsider the fascia’s fundamental role and fascial dysfunction. While the model of excess stiffness has predominated thus far, a complete approach to fascia should also incorporate the reduced stiffness model into daily therapeutic practice. For this latter altered fascial function, the most effective and safest therapeutic approach remains physical exercise. However, significant research on the subject remains necessary before scientists can identify which fascial component - series or parallel - is involved in dysfunctions and, subsequently, which type of physical activity can be used to treat the type of dysfunction.

These fascial training suggestions should not replace muscle strengthening, cardiovascular training, and coordination exercises; rather, they should be a useful addition to a comprehensive training program, particularly to prevent and treat musculoskeletal pathologies.

To build an elastic and injury-resistant fascial network, we must translate the current insights from the dynamically developing fascia research field into practical training programs to encourage physiotherapists, osteopaths, sports trainers, and other movement educators to incorporate the principles presented here and apply them in their specific context.
